# Comparative Molecular Characterization of Resistance and Virulence in *Staphylococcus aureus* from Sewage Effluents and Impacted Marine Outfalls

**DOI:** 10.3390/microorganisms14030585

**Published:** 2026-03-05

**Authors:** Ohud Muslat Alharthy, Amal S. Alswat, Seham Saeed Alzahrani, Monerah S. M. Alqahtani

**Affiliations:** 1 Department of Biotechnology, College of Science, Taif University, P.O. Box 11099, Taif 21944, Saudi Arabia; 2 Biology Department, Faculty of Science, King Khalid University, Abha 61413, Saudi Arabia

**Keywords:** antimicrobial resistance, environmental monitoring, *mec*A gene, methicillin-resistant *Staphylococcus aureus*, virulence genes

## Abstract

Environmental surveillance is important to monitor and mitigate antimicrobial resistance (AMR). In this context, sewage and its marine outfalls remain a hot spot for spreading AMR among pathogens. This study investigated the presence of drug-resistant *Staphylococcus aureus* in sewage effluent and marine sewage outfalls in Saudi Arabia. Water samples were collected from Jeddah’s southern and central marine outfalls and non-impacted sites. The isolates (n = 120) were identified through biochemical tests and MALDI-TOF. Resistance to antibiotics in the isolates was initially screened through phenotypic methods. Species-specific markers and antibiotic resistance genes (ARGs) were amplified through PCR. The presence of ARGs was also quantified in the isolates and in the environment through qPCR. The data indicated a higher prevalence of methicillin-resistant *S. aureus* (MRSA) in sewage effluent (63.3%) compared to marine water (50%). Sewage-borne MRSA exhibited higher resistance to various antibiotics. PCR detection confirmed the presence of *mec*A in MRSA isolates. Virulence genes encoding microbial surface components and recognizing adhesive matrix molecules (MSCRAMMs) were more prevalent in sewage isolates. Particularly, genes responsible for biofilm formation were more prevalent in the isolates from sewage samples. qPCR revealed a higher abundance of *mec*A, *fnb*B and *bbp* in sewage-derived isolates. Statistical analysis confirmed the strong influence of the sewage environment on the prevalence of drug-resistant isolates. Screening of environmental DNA further validated sewage as a reservoir of resistance and virulence determinants. These findings highlight the role of sewage outfalls in disseminating ARGs and virulent *S. aureus* strains, emphasizing the need to improve wastewater treatment and environmental surveillance strategies.

## 1. Introduction

Marine sewage outfalls are meant to export treated or partially treated wastewater beyond the shoreline [1,2]. These are used to manage constraints due to space or public health concerns. Owing to dilution and dispersion effect, these outfalls are justified as means to minimize the contaminant load and human exposure [3]. Yet, some studies indicated alterations in coastal biogeochemistry, benthic habitats and microbial communities, highlighting the effect of effluent beyond the initial mixing zone [4]. Hence, concerns arise in tracing contaminants beyond indicators (e.g., BOD or coliforms). Evidently, the contaminants, such as microplastics, can provide matrix support for the contaminating bacteria, including drug-resistant bacteria, thereby providing a breeding ground for the dissemination of antimicrobial resistance genes (ARG) [5]. The transmission through horizontal gene transfer methods, particularly conjugation and transformation, can cause widespread dissemination of ARGs.

Outfall characteristics and their effects depend on hydrodynamic conditions, discharge volume and treatment levels. Generally, low-energy embayment and large volumetric loads can increase hypoxia risk and the accumulation of contaminants among benthic populations [4,6]. The impacts of these outfalls have been aggravated due to climate-driven problems in coastal stratification. Nonetheless, the targeted surveillance of ARGs and high-risk pathogens is essentially required to minimize public health concerns.

Tracking the presence of microorganisms in marine environments and sewage outfall sites remains a subject of interest. Considering the limitations of traditional methods, molecular techniques have been widely explored in various studies. For instance, Gibb et al. targeted 16S rDNA and 18S rDNA markers to detect the presence of prokaryotes and eukaryotes in eDNA obtained from the Belmont Wastewater Treatment Works (WWTW) ocean outfall in New South Wales, Australia [7], while qPCR-based detection of bacterial species was also carried out earlier using extracted DNA and a newly built device from the samples collected from Monterey Bay, CA, USA [8]. Macena et al. targeted viral DNA and enteric bacteria in their qPCR-based analysis of the marine environment near Brazil [9]. In their studies, Bhikhoo et al. relied on a culture-based technique to investigate microplastic-associated bacteria in a sewage outfall site, but argued the limitation of this technique in contrast to whole-genome sequencing and qPCR-based analysis [5].

Some reports have confirmed the presence of ARGs and drug-resistant bacteria in outfalls by culture-based or molecular techniques [10]. Particularly, the presence of extended spectrum β-lactamases (ESBL)-producing Enterobacterales (including *E. coli* and *Klebsiella* spp.), carbapenem-resistant Enterobacterales (CRE), vancomycin-resistant *Enterococcus* (VRE) and diverse ARG groups (*mec*, *van*, and *bla*_OXA_) has been reported [10,11]. However, there are geographic variations. For instance, in Spain, a surveillance study recovered ESBL-producing *E. coli* and CRE isolates from effluent and the receiving environment, documenting the detectable proportions of these resistant organisms [12]. The researchers argue that the conventional secondary treatment often fails to completely eliminate the high-risk organisms. In North America, the enrichment of ARGs and resistant pathogens was detected by a metagenomics approach [13]; the study reported a greater abundance of these pathogens near long-range coastal outfalls than in distal waters. In Asia and other tropical regions, the prevalence of multidrug-resistant (MDR) bacteria has been described in coastal zones and sediments, with incomplete removal of drug-resistant bacteria affirmed during treatment processes [14].

In addition to Gram-negative bacteria, some Gram-positive bacteria, including *Staphylococcus aureus*, have also been detected in coastal waters, sands and outfall-impacted zones. Although with low frequencies rather than Gram-negative bacteria, methicillin-resistant *S. aureus* (MRSA) has also been found in environmental surveillance programs [15]. The prevalence of this drug-resistant bug has been linked with sewage discharge or stormwater inflows [10,11]. The prevalence of MRSA is perceived to be under-reported, as its isolation and detection require special media that are not routinely used in surveillance programs targeting indicator organisms.

*Staphylococcus* species, including *S. epidermidis*, *S. haemolyticus* and *S. hominis*, have also been isolated from the Red Sea coast in the Jeddah and Jazan regions of Saudi Arabia [16]. The researchers employed a culture-based, as well as a 16S rDNA-based, sequencing approach to demonstrate sewage contamination in coastal water. Among ARGs, those encoding resistance to aminoglycosides, β-lactams, sulfonamides, macrolides, quinolones and tetracyclines with *qnr*, *aac* and *bla* genes were detected at a higher abundance in human-impacted coastal samples. These data indicated the presence of contaminants in coastal sediments across the Saudi coastline [17]. However, most of these studies targeted coagulase-negative Staphylococci (CONS) instead of *S. aureus* or MRSA. The surveillance across the Kingdom of Saudi Arabia has suggested the prevalence of MRSA in hospital as well as community settings [16]. Other studies have also emphasized the two sides of marine microbial fauna as a threat and opportunity, as well as antimicrobial metabolite-producing microbes, and ARG-carrying bacteria have been isolated from coastal areas [18]. However, studies specifically targeting *S. aureus* in this coastline are scarce, and hence, it warrants investigation. In this context, the present study describes the presence of *S. aureus* across the coastline and the detection of particular resistance genes in the isolates.

## 2. Materials and Methods

### 2.1. Sample Collection

**Figure 1:**
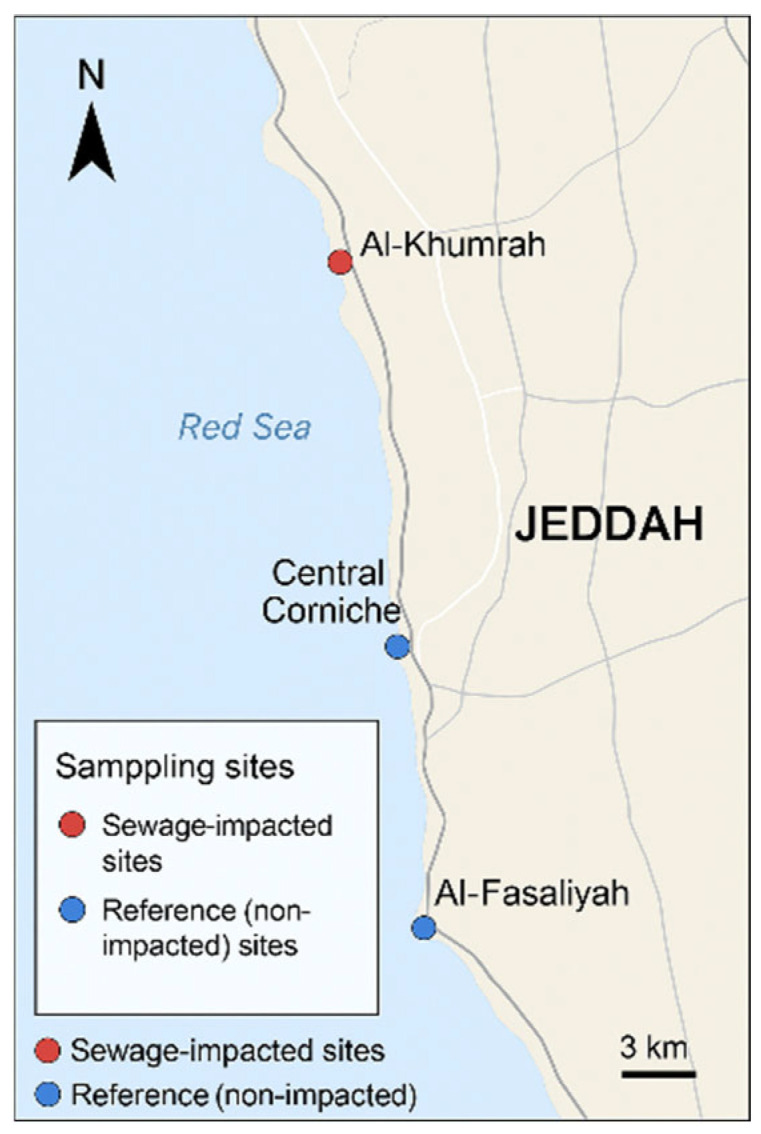
Representation of sampling sites.

Samples were collected from near-shore sites along Red Sea in Saudi Arabia; specifically, Jeddah’s southern and central marine outfalls, adjacent to Al-Khumrah’s southern outfall (21.257° N, 39.112° E), and the central corniche discharge zone (21.492° N, 39.158° E) were selected (Figure 1). For comparison, sampling was also carried out at Al-Fasaliyah (21.482° N, 39.149° E) and central corniche as non-impacted sites (21.500° N, 39.160° E). From each site, surface water (0.5–1.0 m) was collected in sterile amber-colored bottles. Three samples from each site were collected during the month of March 2025. The samples were kept on ice and processed within 6–8 h of the collection. Before processing, water samples were passed through 0.45 μ filters, and the filter membranes were placed on the microbiological media plates and in enrichment broth.

### 2.2. Isolation of S. aureus

An aliquot (100 μL) of the sample from filtered water was spread onto mannitol salt agar (MSA) and Baird-Parker agar (BPA) supplemented with egg yolk tellurite and CHROM agar. Plates were incubated at 37 °C for 24–48 h under aerobic conditions. Typical and presumptive *S. aureus* colonies were subcultured on blood agar to ascertain the hemolysis pattern.

In a parallel experiment, to avoid false negative results due to low abundance of *S. aureus*, the samples (1 mL each) were added to 100 mL of tryptic soy broth (TSB) with 6.5% NaCl and incubated at 35 °C for 18–24 h. After enrichment, 100 μL was streaked onto MSA, BPA and CHROM agar, as stated earlier, and incubated. Presumptive colonies were retained as pure cultures and stored in 70% glycerol at −80 °C.

### 2.3. Identification of S. aureus

The colonies presumptively identified as *S. aureus* were processed for Gram staining, catalase and coagulase tests and biochemical tests through API Staph kit (bioMérieux, Marcy-l’Étoile, France), following manufacturer’s instructions. The identification was further confirmed through MALDI-TOF (Bruker Microflex). Identification score thresholds were applied at score ≥ 2.0 for species-level identification and 1.7–1.99 for genus-level identification.

### 2.4. Antimicrobial Susceptibility Testing (AST)

AST of the confirmed *S. aureus* isolates was performed following Clinical Laboratory Standards Institute (CLSI) guidelines [19]. Initial screening was carried out using disc diffusion method. A bacterial suspension prepared in MHB was adjusted to 0.5 McFarland and spread on MHA plates. The antibiotic discs were placed and the plates were incubated at 37 °C for 18–24 h. Antibiotics included ampicillin, ciprofloxacin, gentamycin, chloramphenicol, ceftriaxone, vancomycin and oxacillin. Zone diameters were interpreted as per CLSI guidelines.

Minimum inhibitory concentrations (MICs) were determined by broth microdilution method, as suggested by CLSI guidelines. *S. aureus* ATCC25923, 29213 and 43300 were used as quality control strains.

### 2.5. Molecular Detection of Resistance Genes

Genomic DNA from the selected isolates was extracted using boiling lysis method. Briefly, two–three isolated colonies from 24 h old cultures on MHA were suspended in 200 μL sterile nuclease-free water, boiled at 100 °C for 10 min and centrifuged at 12,000× *g* for 5 min. The supernatant was transferred to a fresh tube and stored at −20 °C until use. The quality of DNA was assessed by taking absorbance at 260 and 280 nm.

The genomic DNA was sent to Macrogen (Seoul, Repubic of Korea) for PCR amplification. The genomic DNA was used as a template to detect and PCR-amplify the targeted genes, including *mecA, blaZ*, *ermA*, *ermB*, *ermC*, *msrA*, *tetK*, *tetM* and (*aac(6′)-Ie-aph(2″)*), along with some virulence markers, *lukS*, *F-PV* and class I integrin gene (*intI1*). The PCR reaction mixture contained 12.5 μL 2× master mix (Thermofisher, Waltham, MA, USA), 0.5 μM of each primer and 2 μL of template DNA, and the volume was made up with nuclease-free water. PCR conditions included initial denaturation at 94 °C for 5 min, 35 cycles of denaturation at 94 °C for 30 s, annealing at the gene-specific temperature for 30 s (Table 1) and extension at 72 °C for 45 s, followed by final extension at 72 °C for 5 min. PCR products were sent to Macrogen (Seoul, Repubic of Korea) whenever sequencing was required.

qPCR detection was carried out using the sets of primers stated in Table 2. The reactions were carried out in a final volume of 20 μL containing 10 μL of SYBR Green Master Mix (ThermoFisher, Waltham, MA, USA), 0.5 μL of each forward and reverse primer, 2 μL of template DNA and nuclease-free water to volume. Cycling conditions included initial denaturation at 95 °C for 3 min, followed by 40 cycles of 95 °C for 10 s and 60 °C for 30 s. A melting curve analysis (65–95 °C) was included to verify amplicon specificity. Gene copy numbers were calculated from standard curves generated using 10-fold serial dilutions of purified amplicons. Relative gene abundance was normalized to *fem*A and expressed as gene copies per ng of genomic DNA. Negative controls were included in qPCR analyses.

### 2.6. Statistical Analysis

The data were recorded in MS Excel and exported to SPSS version 30. The prevalence of *S. aureus* and resistance markers was calculated at 95% confidence level with *p-*value < 0.05. Categorical variables (e.g., presence/absence of resistance and genes) were compared between sewage and marine isolates using Chi-square (χ^2^) test. Fisher’s exact test was applied when the expected cell counts were less than five. Bonferroni correction was applied to analyze multiple comparisons. The adjusted significance threshold was determined using conventional alpha level.

## 3. Results

### 3.1. Distribution of S. aureus and MRSA in Environmental Sources

A total of 120 isolates identified as *S. aureus* were recovered: 60 from marine water and 60 from sewage effluents (Table 3). The isolates were analyzed for their methicillin-resistant status. Overall, the prevalence of 56.7% of isolates was confirmed as MRSA. However, the two environments differed in the prevalence of MRSA isolates. A higher proportion of MRSA (63.3%) was observed from the sewage source compared to the marine water (50%). It shows the source of MRSA in the environment and a higher burden of antibiotics in the effluent. The results were validated by considering the isolates from non-impacted sites. No isolate was found to be MRSA from these two sites, and hence, analysis of these isolates did not proceed further. Nonetheless, it affirmed that the sewage was the primary source of MRSA in the marine water. Any intrusion from distant waters may have been minimized, considering the unfavorable marine environment for the *S. aureus* isolates.

### 3.2. Antibiotic Susceptibility Profiles of the Isolates

**Figure 2:**
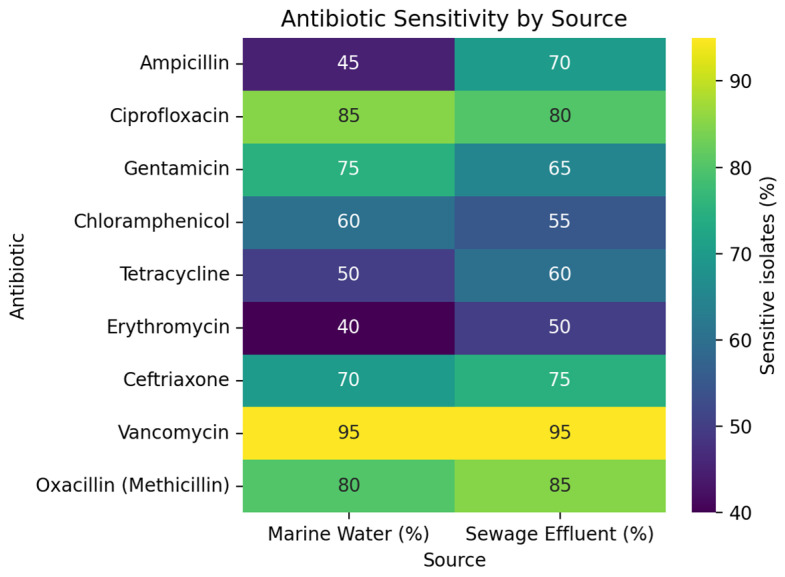
Heat map illustrating the percentage of *S. aureus* isolates sensitive to various antibiotics and comparing isolates from marine and sewage environments.

The isolates from both the marine water and sewage effluent exhibited high levels of susceptibility (95%) towards vancomycin, highlighting the preserved efficacy and low dissemination of this antibiotic (Figure 2). Ciprofloxacin demonstrated 85% susceptibility in marine isolates but 80% in sewage-borne isolates. A similar trend was observed for gentamicin, indicating the presence of more resistant isolates in the effluent. The responses against b-lactams showed higher variability, as the isolates from sewage were more resistant than the isolates from marine sources. Third-generation cephalosporin (ceftriaxone) demonstrated intermediate to high activity against both types of isolates, while the older, broad-spectrum antibiotics displayed comparatively reduced performance.

### 3.3. Comparative Resistance Among MRSA Isolates

The subset population of MRSA (n = 68) was separately analyzed for its resistance profile. MRSA from sewage showed higher resistance across multiple antibiotic classes (Table 4), affirming the higher antibiotic burden in sewage effluent. With *p* < 0.05, sewage-borne MRSA exhibited higher resistance to ciprofloxacin. Although these isolates were more resistant to ceftriaxone, gentamicin, erythromycin and vancomycin than the isolates from the marine environment, the difference was not statistically significant. These findings highlight the role of sewage as a conducive environment for AMR spread.

### 3.4. Prevalence of mecA, femA and MSCRAMMs Genes Among the Isolates

The *mec*A gene was detected in 30 and 38 isolates from marine and sewage sources (Table 5), corroborating the phenotypic detection of MRSA, while the *fem*A gene, which is usually used as a species-specific marker, was detected in all of the isolates.

The prevalence of virulence markers, MSCRAMMs, was also analyzed separately in the isolates from two origins (Table 6). Adhesion-related genes (*clf*A, *fnb*A and *cna*) were detected at higher frequencies, indicating the conservation of these genes in the environmental *S. aureus* isolates. However, *bbp* and *fib* were more prevalent in the isolates from the sewage source than in the marine water. It indicated that the sewage-borne isolates acquired genes encoding biofilm synthesis, enabling them to survive in nutrient-dense environments.

### 3.5. Quantitative Analysis of Resistance and Virulence Determinants

Copy numbers of *mec*A, *fem*A, *fnb*B and *bbp* genes were quantified using qPCR (Table 7). Except for *fem*A, all the other target genes were found with high burdens. Sewage isolates carried more than double the copies of *mec*A per ng of DNA (2.8 × 10^3^ ± 9.0 × 10^2^) compared to the marine *S. aureus* isolates (1.3 × 10^3^ ± 4.0 × 10^2^). This difference indicates stronger selective pressure for methicillin resistance in the isolates of sewage origin.

Likewise, both *fnb*B and *bbp* were significantly more abundant in sewage isolates (1.4 × 10^3^ ± 5.0 × 10^2^ and 1.6 × 10^3^ ± 6.0 × 10^2^ copies/ng DNA, respectively) than in marine isolates (8.0 × 10^2^ ± 3.0 × 10^2^ and 7.0 × 10^2^ ± 2.5 × 10^2^). This finding aligned with the conventional PCR findings and underscores the tendency of sewage-borne isolates to harbor more adhesion- and persistence-related markers.

**Figure 3:**
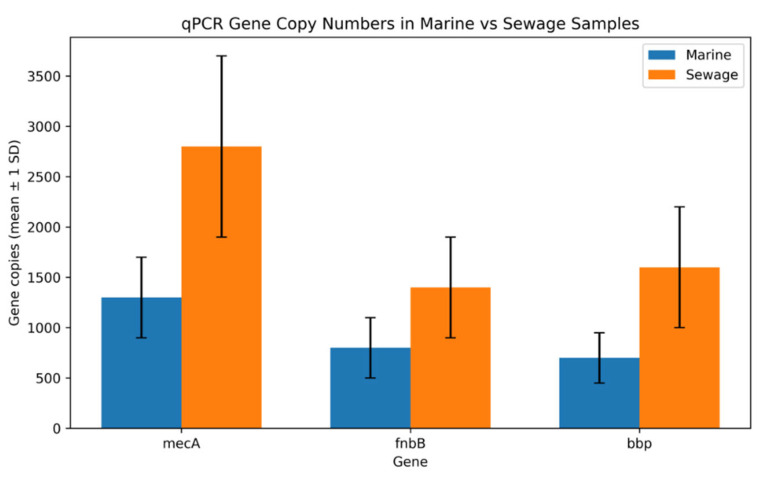
Quantitative detection of *mec*A, *fnb*B and *bbp* genes in *S. aureus* isolates from marine and sewage environments. Bars represent mean gene copy numbers per ng of genomic DNA (log_10_ scale), as determined by qPCR (n = 60 persource). Error bars indicate standard deviation.

These patterns have been represented in Figure 3, where a pronounced increase in *mec*A, *fnb*B and *bbp* abundance among sewage isolates is evident on a log_10_ scale.

Furthermore, Bonferroni corrections were applied to obtain insights from the data, which showed a 2.15-fold increase and a higher copy number of the *mec*A gene in sewage compared to the marine water (Table S1). This difference was associated with a large effect size (Cohen’s d ~2.15) and was statistically significant, while for *fnb*B and *bbp*, the copy numbers were 1.75-fold and 2.29-fold higher in sewage than in the marine water, with a Cohen’s d of 1.46 and 1.96, respectively. It further strengthened our finding regarding the enrichment of the sewage environment with AMR-related genes.

### 3.6. Detection of Resistance and Virulence Genes in Environmental DNA

**Figure 4:**
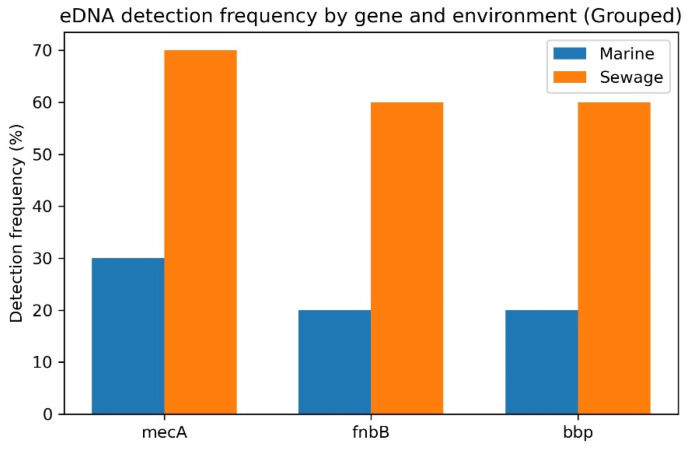
Detection frequency of *mec*A, *fnb*B and *bbp* genes in environmental DNA (eDNA) sampled from marine water and sewage effluent. Percentages represent proportions of positive samples out of 10 analyzed per source.

In addition to the isolates, environmental DNA was extracted and screened for *mec*A, *fnb*B and *bbp* markers across sewage and marine samples (Figure 4). A higher level of detection of these markers was noted from sewage sources (60–70%) compared to the marine water (20–30%). These results further validate the finding of higher resistance in the isolates from sewage than from the marine environment.

## 4. Discussion

AMR poses a great threat to health sectors worldwide, and hence, tracking its sources is crucial to mitigate AMR spread [20,21,22]. For community-acquired infections, drinking water has been linked with the transmission of pathogenic bacteria [23]. Sewage has long been recognized as a breeding ground of drug-resistant bacteria [24]. Considering the variable sanitation-related policies across the globe and poor hygiene conditions, particularly in developing countries, sewage acts as a potential source of the dissemination of drug-resistant bacteria to communities [25]. Indeed, water, sanitation and hygiene (WASH) have been recognized as the most important factor in the spread of community-acquired infections [24,25]. However, extensive investigations on sewage as a source of transmission of non-enteric pathogens are still lacking [26]. Our findings on the higher prevalence (63.3%) of *S. aureus* from sewage sources than the marine water (50%) corroborated some earlier surveillance studies [27,28]. It highlights wastewater as a major reservoir of antibiotic-resistant bacteria due to the continuous influx of residues from clinical set-ups, pharmaceutical industries and communities [29]. Sewage treatment plants (STPs) have been identified for their shortcomings in removing pharmaceuticals from the wastewater [30]. This persistence and accumulation of active pharmaceuticals in sewage creates a selective pressure on the microbial communities and thereby sensitive bacteria are removed, and the ecosystem becomes more growth-supporting for the drug-resistant bacteria [30,31]. The culture-based technique to detect *S. aureus* in the sample provides high confidence in confirming the presence of viable organisms and, hence, indicates the shortcomings of the treatment processes. The next-generation sequencing (NGS)-based technique, although more sensitive, cannot distinguish between viable and non-viable organisms.

The presence of *mec*A-positive isolates has also been reported in untreated and treated sewage [32,33], suggesting a selective pressure exerted by the drug residues. The *mec*A gene has been recognized as a universal marker to detect MRSA, regardless of its origin from community or hospital settings [34]. The dilution factor in marine water, increased ultraviolet exposure, salinity stress and nutrient availability may also have impacts on the lower prevalence of drug-resistant *S. aureus* isolates in this environment [35,36]. Nonetheless, the higher number of isolates near sewage outfalls highlights an environmental threat and has also been reported earlier from similar sites in other countries, including China and Spain [12,37,38]. This global trend across the south and north highlights the importance of modifications in wastewater treatment processes to effectively eliminate pathogenic bacteria.

The consistently higher drug resistance in the isolates detected, particularly in the sewage-borne isolates, reflected the presence of multiple ARGs in the isolates. Such a co-occurrence of ARGs reflects the enrichment of mobile genetic elements and other driving factors in the bacteria thriving in the sewage ecosystem [39,40]. However, the isolates demonstrated low resistance to clindamycin and chloramphenicol across both environments, indicating that these agents remain less widespread in such environments. Contrary to this study, consistently higher levels of clindamycin were reported earlier in sewage samples [41]. It may be attributed to local antibiotic prescription practices. The preserved efficacy of linezolid and rifampin further suggests a limited dissemination of these critical antimicrobials, which may be due to fewer prescriptions. The conserved efficacy of oxazolidinone has been reported earlier, owing to its uncommon use outside hospital settings [42]. Indeed, the efficacy of this antibiotic maintenance is owed to its reserved use. The marked divergence in the resistance patterns in marine isolates from the sewage isolates, as given in the heat map, presents the differential antibiotic stress in the two environments.

This difference in the AMR status of the isolates from two habitats was also confirmed by statistical analysis. Sewage-borne MRSA isolates demonstrated significantly higher resistance to several antibiotics, such as ceftaroline, ciprofloxacin, trimethoprim-sulfamethoxazole and chloramphenicol. This can be attributed to the presence of a mixture of antibiotics in sewage, as described earlier [35,43]. In contrast, the resistance of gentamicin and tetracycline in sewage-derived MRSA was not statistically significant. This can be attributed to variable exposure histories and a lack of horizontal gene transfer for these markers [44]. Regarding the presence of specific genes, *fem*A was found universally in all the isolates, emphasizing the reliability of this marker for species confirmation [39], while *mec*A was detected in 30 marine- and 38 sewage-borne isolates, corroborating the phenotypic assay. The agreement between phenotypic resistance and qPCR-based analysis supported the reliability of the methodology employed in this study.

Virulence-associated MSCRAMMs were also found to be different in the isolates from the two environments. Conserved adhesion genes, such as *clf*A, *fnb*A and *cna*, were detected at higher frequencies in the isolates regardless of their origin. These genes have been found in various MRSA and methicillin-sensitive *S. aureus* isolates from patients [45] and from sewage [46], indicating the role of these genes in environmental persistence and attachment to the particulate materials. However, biofilm-related genes, including *bbp, fnb*P and *fib*, were more common in sewage-borne MRSA, reflecting their enhanced capacity for surface colonization. This feature in sewage isolates has been associated with nutrient-rich but stress-intensive conditions in the sewage environment [23]. Although whole-genome-based analysis could provide deeper insights into the individual isolates, the targeted approach adopted in this study demonstrated differential distribution of clinically relevant markers between the two habitats.

The qPCR-based analysis further affirmed selective pressure on sewage isolates, as *mec*A burden was more than twice as high in these isolates compared to the marine counterparts. Likewise, the biofilm markers were more abundant in sewage isolates, reinforcing the idea that the wastewater environment selects for isolates equipped with enhanced adhesion and persistence capabilities. The strong effect sizes observed after Bonferroni correction underscore the robustness of these differences and corroborate the wastewater genomic surveys, where resistance and adhesion determinants co-present at elevated levels [47,48,49]. It is noteworthy that although marine isolates harbored a low burden of resistance and virulence genes, these were not free of these markers and highlight the potential of marine environments and seafood as reservoirs of ARG-containing pathogens.

The findings were further enriched by screening environmental DNA, which showed sewage as a concentrated environment of ARGs and virulence determinants. The widespread presence of *mec*A, *fnb*B and *bbp* in sewage environments suggests that these genes are widespread within the broader microbial and extracellular DNA pool. Such enrichment of resistance and adhesion markers in wastewater systems has been reported earlier and linked with cell lysis, biofilm dispersal and horizontal gene transfer [50,51,52]. It is worth noting that harsh marine environments can cause damage to DNA, limiting gene transfer processes. Indeed, ultraviolet exposure, high salinity and hydrodynamic dispersal pose challenges to the bacteria and dispersal of ARGs [53,54]. Nonetheless, the detection of these genes in marine environments poses a serious threat, as these can be transferred to indigenous marine bacteria and can broaden the ecological footprints of resistance. Hence, it provides intriguing evidence to modify the existing environmental surveillance programs and wastewater treatment processes. Future studies integrating targeted qPCR techniques (used in this study) with technologically advanced techniques, such as NGS, would provide a holistic view. It will combine functional validation with whole-resistome mapping, thereby enhancing risk assessment and policy-driven environmental AMR monitoring.

The findings of this study should be considered in the light of certain limitations. This study provides data from a particular geographic region and may not be valid for other regions. Moreover, the sampling was carried out only once, and seasonal changes were not studied. The detailed genomic characterization of the isolates using whole-genome sequencing was not carried out, and hence, the presence of other resistance markers cannot be ruled out.

## 5. Conclusions

The findings of this study demonstrated the higher prevalence of methicillin-resistant *Staphylococcus aureus* (MRSA) in sewage than in marine water. Moreover, sewage-derived MRSA exhibited a higher resistance to various antibiotics that can be linked to the presence of pharmaceutical residues in the sewage. qPCR quantification of eDNA affirmed the presence of virulence and resistance genes in sewage samples, which can be attributed to the higher number of sewage-borne isolates carrying these markers. It highlighted the role of sewage as a breeding ground for the emergence and dissemination of drug-resistant bacteria. Future studies encompassing molecular assays to track the clonal dissemination among the two environments are required to strengthen the environmental surveillance programs.

## Figures and Tables

**Table 1:** PCR primer sets and annealing temperatures used for the detection of antibiotic-resistance and virulence genes in *S. aureus* isolates.

Target Gene	Primer Sequence	Amplicon Size (bp)	Annealing Temperature (°C)
*mec*A	F: AAAATCGATGGTAAAGGTTGG R:AGTTCTGCAGTACCGGATTTG	310	55
*bla*Z	F: ACTTCAACACCTGCTT R: TGACCACTTTTATCAGCAACC	173	54
*erm*A	F: AAGCGGGTTAAGGAGTTGTTGA R: AGGGTTTCCTTCCTTAGCG	190	52
*erm*B	F: GAAGGATAACTCTAAACCTCTA R: TTTTACTCTTTGAATCGGTTA	142	53
*erm*C	F: TCATGTGAAAAGGTATCAAAAC R: CTTTCAATGTAAAGATTTCCTA	299	52
*msr*A	F: GGCTATCATTTTGATCAAAGACC R: AGATTATCAGCATTATTTGTGG	163	53
*tet*K	F: TTAGGTTCTTGATGTTGCGG R: TGATTGAGAGCTTAACTCGG	360	55
*tet*M	F: GTGGACAAAGGTACAACGAG R: CGGTAAAGTTCGTCCGTGTA	406	54
*aac(6)-le-aph(2)*	F: CTTGATGATGGCTGGTTTGGC R: CCTCGATGAACAAGCTTTCCG	369	58
*luk*S/F-PV	F: ATCATTAGGTAAAATGTCTGGACATGATCCA R: GCATCAAGTGTATTGGATAGCAAAAGC	433	55
Intl1	F: GGCATCCAAGCAGCAAG R: AAGCAGACTTGACCTGAT	280	56

**Table 2:** Primer sequences used for the qPCR detection of antibiotic-resistant genes.

Gene	Primers
*mec*A	F: TCCAGACAACAACCTC R: TTTCTTGGGTGTGACTTT
*fem*A	F: ACGAACAACTTTGCGAA R: TGAGCTTATTGGTGTTGTCC
*fnb*B	F: GATCAAGACAACGATAA R: CATTGCCGTTGAGAATGTTG
*bbp*	F: CAAGGTACTGGTGGACAAGA R: TTGGTCTGTTTGGTAACCAG

**Table 3:** Sampling sources and distribution of methicillin-resistant *Staphylococcus aureus* (MRSA) and methicillin-sensitive *S. aureus* (MSSA).

Source	Total Isolates (n)	MRSA (*mec*A+)	MSSA (*mec*A−)	MRSA (%)
Marine water (impacted sites)	60	30	30	50.0
Sewage effluent	60	38	22	63.3
Marine water (non-impacted sites)	13	Nil	13	Nil
Total	133	68	65	51.1

**Table 4:** Comparative antibiotic resistance among methicillin-resistant *S. aureus* (MRSA) isolates recovered from marine water and sewage effluent.

Antibiotics	Resistant MRSA (%) From		*p-*Value *
	Marine Water	Sewage Effluent	
Ampicillin	100.0	100.0	-
Ceftriaxone	83.3	89.4	0.11
Gentamicin	30.0	39.4	0.18
Erythromycin	53.3	63.1	0.09
Tetracycline	46.6	52.6	0.22
Ciprofloxacin	46.6	63.2	0.03
Vancomycin	6.6	5.2	0.11
Oxacillin	100.0	100.0	-
Chloramphenicol	13.3	28.9	0.05

* *p-*value < 0.05 was considered significant.

**Table 5:** Prevalence of *mec*A and *fem*A genes among *S. aureus* isolates from marine water and sewage effluents.

Gene	Positive Isolates (%)		Total Positive Isolates (%)
	Marine (n = 60)	Sewage Effluent (n = 60)	
*mec*A	30 (50.0%)	38 (63.3%)	68 (56.7%)
*fem*A	60 (100%)	60 (100%)	120 (100%)

**Table 6:** Distribution of MSCRAMMs genes among *S. aureus* isolates from marine water and sewage effluents.

Gene	Function	Marine Isolates (%)	Positive Sewage Isolates (%)
*clf*A	Fibrinogen binding	98.3	100
*clf*B	Fibrinogen binding	93.3	95.0
*fnb*A	Fibronectin binding	96.6	98.3
*fnb*B	Fibronectin binding (biofilm)	73.3	85.0
*bbp*	Bone sialoprotein binding	65.0	78.3
*cna*	Collagen binding	91.6	95.0
*eno*	Enolase (laminin binding)	71.6	76.6
*fib*	Fibrinogen interaction	68.3	81.6

**Table 7:** qPCR quantification of resistance (*mec*A) and virulence (*fnb*B, *bbp*) genes in *S. aureus* isolates from marine water and sewage effluent.

Target Gene	Copies/ng DNA in (Mean ± SD)		Fold Change Sewage/Marine	*p-*Value
	Marine Isolates	Sewage Isolates		
*mec*A	1.3 × 10^3^ (±4.0 × 10^2^)	2.8 × 10^3^ (±9.0 × 10^2^)	2.15	<0.001
*fem*A	9.8 × 10^3^ (±1.1 × 10^3^)	1.0 × 10^4^ (±1.2 × 10^3^)	1.04	0.26
*fnb*B	8.0 × 10^2^ (±3.0 × 10^2^)	1.4 × 10^3^ (±5.0 × 10^2^)	1.75	<0.001
*bbp*	7.0 × 10^2^ (±2.5 × 10^2^)	1.6 × 10^3^ (±6.0 × 10^2^)	2.29	<0.001

## Data Availability

Data are provided in the manuscript and associated Supplementary Materials.

## Supplementary Materials

The following supporting information can be downloaded at https://www.mdpi.com/article/10.3390/microorganisms14030585/s1. Table S1: Bonferroni analysis of presence of *mec*A, *fnb*B and *bbp* in the isolates from sewage and marine water.
